# Correlations Between Carbon Structure and Properties by XRD and Raman Structural Studies During Coke Formation in Various Rank Coals

**DOI:** 10.3390/ma19010168

**Published:** 2026-01-02

**Authors:** Lu Tian, Jinxiao Dou, Xingxing Chen, Jianglong Yu

**Affiliations:** 1 Key Laboratory of Advanced Coal and Coking Technology of Liaoning Province, School of Chemical Engineering, University of Science and Technology Liaoning, Anshan 114051, China; 320163300148@ustl.edu.cn (L.T.); xingchenstar79@163.com (X.C.); 2 Suzhou Industrial Park Monash Research Institute of Science and Technology, Southeast University-Monash University Joint Graduate School, Suzhou 215123, China

**Keywords:** bituminous coal, carbon structure, coal rank, coke properties

## Abstract

The structure and properties of coke are of significant importance in the metallurgical industry. Coke samples were prepared from different bituminous coals at varying temperatures using a one-sided heating furnace. The evolution of carbon structure during the coking process was investigated by X-ray diffraction (XRD) and Raman spectroscopy. The correlations between carbon structure parameters and the properties of the coal and coke were investigated during coke formation. The results indicated that with increasing temperature, the values of L_a_, L_c_, N, *n*, and f_a_ were increased, while the d_002_ values decreased. The L_a_/L_c_ ratio was expanded twice more than raw coal due to condensation and cross-linking reactions, indicating compaction of the carbon structure and the formation of larger aromatic units. A negative correlation was observed between L_c_ and the Coke Reactivity Index (CRI), whereas a positive correlation was found between L_a_ and Coke Strength after Reaction (CSR), which mean that coke properties improve with increasing L_c_. Specifically, when L_c_ exceeds 2.4 nm, and L_a_ lies between 5 and 5.5 nm, the coke exhibits higher quality. The quality of coke is strongly affected by the structural evolution of carbon during the coal coking process.

## 1. Introduction

The various ranks of bituminous coals serve as essential raw materials for coking, producing coke utilized in the metallurgical industry. In metallurgical processes, coal plays a critical role not only as a fuel but also as a burden material in blast furnaces, reducing agent, and carbonization medium for reduced iron. Understanding the development of coke’s properties and its carbon structure is therefore of considerable importance [1,2,3]. To assess the suitability of valuable bituminous coals for coking, investigations into their carbon structural characteristics and coking behavior are indispensable [4,5,6]. Such studies offer valuable insights for evaluating and predicting the influence of coal’s macromolecular structure on coke formation, which holds significant implications for advancing integrated coal utilization technologies [1,6,7].

In recent years, several studies have documented alterations in coal properties during the coking process. The pressure generated by coke formation has been shown to influence the evolution of the carbon structure under coking conditions [8]. For instance, Lee et al. [8] employed a 4 kg laboratory-scale coke oven to measure internal gas pressure (IGP) and temperature distributions at five distinct locations under simulated coke heating conditions. Samples of semi-coke and coke were extracted during carbonization to examine physical transitions and fissure development via micro-CT analysis. Their results indicated that the extent of fracture development affected the maximum IGP; insufficient fracture formation inhibited the release of volatile matter, leading to peak pressure at the center of the coke oven. The thermoplastic characteristics of different coal zones during carbonization were correlated with coal properties [9]. This relationship was established using penetrometer-resistance curves derived from thermogravimetry–plastometer–swelling pressure apparatus, applied to seventeen coking coals of varying rank.

The evolution of carbon structures in coals during the coking process has been extensively investigated. Chen et al. [10] demonstrated that coal rank exerts a significant influence on the transformation of cross-linking structures and the evolution of aromatic lamellae during coke formation. Specifically, C–C bonds in semi-coke are established, and aromatic clusters grow through enhanced crosslinking, polymerization, and condensation, resulting in increased structural ordering at elevated temperatures. Another study [11] examined the development of carbon structures during the coking of coals at various metamorphic stages, revealing that the petrographic characteristics of the coals affect the transformation of carbon structures during coking. Using X-ray photoelectron spectroscopy (XPS) and high-resolution transmission electron microscopy (HRTEM), Xu et al. [12] investigated the evolution of carbon structures in five coking coals of different ranks across the temperature range of 600–1000 °C. Their findings indicated that cross-linking structures and aliphatic cross-links convert into graphitized carbon structures during coking, accompanied by the condensation of aromatic rings. This process leads to an increase in the size of aromatic layers and promotes their flattening. Compared with techniques such as ^13^C NMR, XPS, TEM, and FTIR, X-ray diffraction (XRD) offers a relatively simple and cost-effective approach for examining ordered packing in coal. Both XRD and Raman spectroscopy are widely employed to determine carbon structure parameters in coals, including crystallite sizes (L_a_, L_c_), interlayer spacing (d_002_), and aromaticity (f_a_).

XRD analysis has been established as a methodological approach for estimating the stacking structure, degree of order, interlayer spacing (d_002_), and microcrystalline dimensions (L_c_/L_a_) of carbonaceous materials in coal, thereby providing a means for evaluating their structural parameters [13,14,15,16]. Related studies have further correlated these structural parameters with coke properties [6,17]. Wei et al. [17] investigated the characteristics and formation process of coke to elucidate the mechanism of quality improvement via hot tamping coking. Their findings indicate that optimal tamping temperature and holding time promote higher semi-coke permeability and reduce volatile retention, resulting in high-quality coke with a lower Coke Reactivity Index (CRI) and a more compact pore structure. Furthermore, reduced CRI, lower porosity, thicker pore walls, and improved homogeneity contribute to enhanced Coke Strength after Reaction (CSR). Despite these advances, the relationship between coke properties (CRI and CSR) and the evolution of coke carbon structure remains insufficiently understood. In particular, the transformation of chemical and carbon structures throughout the plastic layer, semi-coke, and coke stages significantly influences the final structure and properties of coke.

The carbon structure and quality of coke are profoundly affected by the rank of coking coal. Understanding the transformation of coal into coke and the characteristics of its carbon structure is crucial. Given the complexity of coal, it is necessary to analyze multiple samples to clarify general trends. Therefore, eleven Chinese bituminous coals with different coalification degrees were chosen in this study. In addition, to gain a comprehensive understanding of the structural evolution of coal to coke and the correlation between coal properties and coke structure, results from XRD and Raman analyses were compared to validate their consistency across different ranks of bituminous coals and their corresponding cokes. The obtained carbon structural parameters will provide a foundation for advancing the study of bituminous coal coking. These data will further support subsequent investigations of the properties of cokes derived from various bituminous coals within coking systems, as well as studies on the molecular structures of coals.

## 2. Materials and Methods

### 2.1. Coal Selection and Analysis

Eleven Chinese bituminous coals with different coalification degrees were chosen in this study, including gas coal (YZX), 1/3 coking coal (PJX), fat coals (XX and WG), coking coals (GYT, ML, MT, LQ, QLS and TL), and lean coal (HBB). The proximate analysis, ultimate analysis, and coal properties are shown in Table 1, in accordance with the relevant Chinese standards (GB/T 212-2008 [18], GB/T 6948-2008 [19], GB/T 4000-2017 [20] and GB/T 1996-2017) [21]. The coal rank, characterized by the mean maximum vitrinite reflectance (R_o max_), covered a broad range from 0.744% to 1.831%. Based on R_o max_ and the volatile matter (V_daf_) index, the coal samples were classified into five categories, as summarized in Table 1. The structural transformation of coke during carbonization significantly influences its properties, notably the Coke Reactivity Index (CRI) and Coke Strength after Reaction (CSR). The selection of coals, determination of economically viable blending ratios, and regulation of coke quality—based on a systematic understanding of coking coal properties—have consistently been key research foci in the global coal blending and coking industry. A thorough understanding of coking coal properties entails not only quantifying the values of relevant indicators at different levels but also evaluating their practical feasibility in industrial applications.

This methodology demonstrates high reliability and can be effectively employed to produce coke with consistent quality. Nevertheless, its widespread implementation faces challenges due to the scarcity of high-quality coking and fat coal resources in China.

### 2.2. Sample Preparation and Method

**Figure 1:**
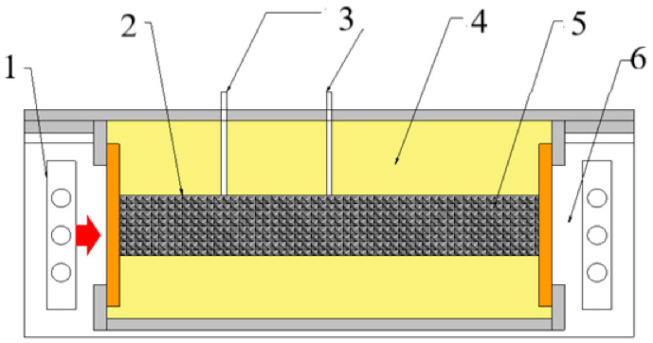
Experimental setup of the coking reactor in a lab-scale coke oven (1. heating elements, 2. quartz reactor of coal charge, 3. thermocouple, 4. insulation, 5. sample, and 6. furnace).

**Figure 2:**
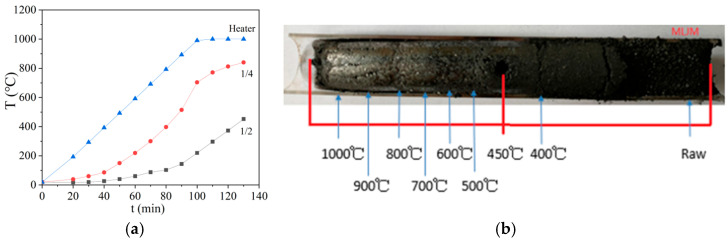
Schematic of quartz tube showing temperature gradient of coal samples and locations at different stages of carbonization: (**a**) temperature distribution of sample in different locations in the reactor; (**b**) the temperature changes of heating part, 1/2, and 1/4 position with time.

The coking experimental setup and sample preparation during the coking process are shown in Figure 1. The samples were placed into a quartz reactor (inner diameter was 20 mm, length was 170 mm, and density was 800 kg/m^3^) and set in the furnace. The furnace was heated from one side in order to simulate one-sided heat transfer from the heating wall. The heating conditions were 10 °C/min from room temperature to 1000 °C and 30 min in a confined space. The center of the sample was 450 °C when the run stopped. The temperature distribution of samples in different locations in the reactor is shown in Figure 2a. The heating rate of samples was 3 °C/min, and it was kept the same as the industry device [1]. The raw sample and the different temperature semi-cokes and coke (one sample is shown in Figure 2b) were prepared for carbon structure analysis.

### 2.3. Analysis of Coal Samples

#### 2.3.1. Thermogravimetric Analysis (TG) Analysis Method

Thermogravimetric (TG) analysis of the coal samples was conducted using a STA 449 F3 thermogravimetric analyzer (Netzsch, Selb, Germany). For each experiment, approximately 40 mg of raw coal was heated from ambient temperature to 1000 °C at a constant heating rate of 10 °C/min under a continuous argon flow of 60 mL/min to maintain an inert atmosphere. The TG and derivative thermogravimetry (DTG) curves were analyzed to characterize the thermal properties of the coal.

#### 2.3.2. FTIR Analysis Method

FTIR analysis was performed using a Nicolet IS 50 Fourier transform infrared spectrometer (Thermo Fisher Scientific Inc., Waltham, MA, USA). To semi-quantitatively compare the functional groups present in different ranks of bituminous coal, samples were subjected to FTIR spectroscopy. Before measurements, a background spectrum was tested by scanning pure KBr powder. Subsequently, each coal sample was evenly spread to a thickness of approximately 2 mm in a sample cell placed over SiO_2_ powder. The spectra were recorded in the wavenumber range from 4000 to 650 cm^−1^ at a resolution of 4 cm^−1^ and 32 scans by a liquid nitrogen-cooled mercury–cadmium–telluride (MCT) detector. The final infrared spectrograms were obtained following background correction.

#### 2.3.3. XRD Analysis of Samples

An XRD instrument (Ultima IV, Rigaku, Tokyo, Japan) with Cu radiation (λ = 0.15418 nm) was used to analyze the carbon structure of coals, semi-cokes, and cokes. The samples were measured with a scan speed of 2°/min from 10 to 70° and a step size of 0.02°. The voltage was 40 kV and the current was 40 mA.

**Figure 3:**
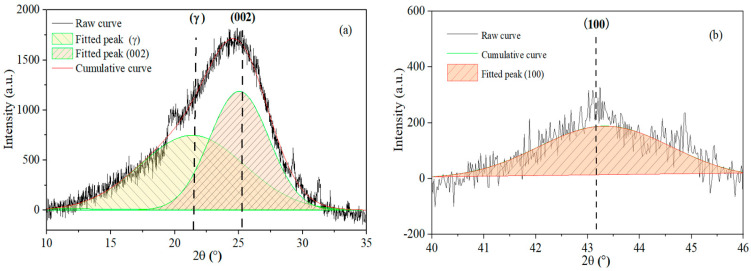
The curve-fitted spectra of samples (YZX 900 °C) on the carbon structure from XRD analysis: (**a**) peak fitting of the 002 and γ bands (10–35°); (**b**) peak fitting of the 100 bands (40–46°).

Carbon structural parameters, including crystallite lattice height (L_c_), lattice diameter (L_a_), aromatic layer spacing (d_002_), the number of aromatic layers (N), stacking layer number (*n*) and aromaticity (f_a_) were calculated by Equations (1)–(6) [7,22,23,24]. Peak analysis was performed using Origin 2021 and employing Gaussian band fitting. The peak centers (θ) and full widths at half maximum (FWHM) for the γ, 002, and 100 bands were derived from the fitting results. The curve-fitted spectra of the sample (YZX 900 °C) on the carbon structure (10–35° and 40–46°) from the XRD analysis were shown in Figure 3.

(1) $$L_{c}=\frac{0.89\lambda }{{\mathrm{FWHM}}_{002\;}\times \;\cos\theta_{002}}$$

(2) $$L_{a}=\frac{1.84\lambda }{{\mathrm{FWHM}}_{100\;}\times \;\cos\theta_{100}}$$

(3) $$d_{002\;}=\frac{\lambda }{2\sin\theta_{002}}$$

(4) $$N=\frac{L_{c}}{d_{002}\;}+1$$

(5) $$n=0.32\;\times \;N^{2}$$

(6) $$f_{a}=\frac{A_{002}}{A_{002\;}+A_{\gamma }}$$

where λ is the wavelength of the X-ray (0.15406 nm) and θ_002_ and θ_100_ are the peak positions of the 002 and 100 bands, respectively. FWHM_002_ and FWHM_100_ are the FWHM of the two bands, respectively. A_002_ and A_γ_ are the integrated areas of the 002 and γ bands, respectively. The Coefficients of Determination (R^2^) were above 0.95.

#### 2.3.4. Raman Analysis of Samples

Raman spectroscopy of the samples was performed using a XploRA PLUS laser Raman spectrometer (Horiba, Loos, France) to evaluate the carbon crystallinity of the coals, semi-cokes, and cokes. A Diode-Pumped Solid-State (DPSS) laser with a wavelength of 532 nm was employed under the following measurement conditions: a diffraction grating of 600 gr/mm, a 50× objective, and a laser power of 1 mW. Spectra were collected in the range of 800–2000 cm^−1^ with an accumulation time of 30 s and 4 accumulations per spectrum, resulting in a total acquisition time of 120 s per scan. In the Raman spectra, the G band corresponded to the graphite-like ordered structure, while the D band reflected structural disorder which was characteristic of defective carbon materials. Each spectrum was deconvoluted into constituent bands associated with different carbon structures. The assignments of the Raman bands for the coal samples are summarized in Table 2 [4,24,25,26].

## 3. Results and Analysis

### 3.1. Analysis of Different Coals During Pyrolysis

#### 3.1.1. TG Analysis

**Figure 4:**
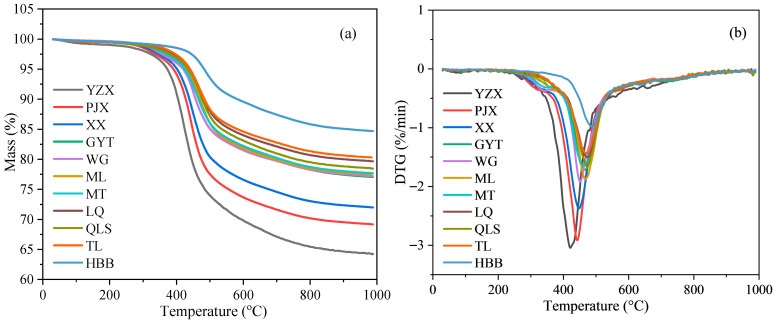
TG–DTG analysis of different coals during pyrolysis: (**a**) TG curves; (**b**) DTG curves.

The pyrolysis performance of raw coal samples was analyzed using TGA. The TG and DTG curves of different coals during pyrolysis are shown in Figure 4.

It can be seen that the total weight losses of raw coals during pyrolysis were decreased with increasing coal rank. The total weight losses of different coals ranging from low rank to high rank were 35.7%, 30.8%, 28.0%, 22.9%, 22.8%, 22.5%, 22.3%, 20.3%, 21.5%, 19.7%, and 15.3%. Consistent with its high volatile matter content, YZX coal exhibited the greatest weight loss, whereas HBB coal, with the lowest volatile matter, showed the least weight loss. This trend was consistent with the volatile matter content of the coals.

Below 300 °C, the decomposition of raw coals was attributed to the removal of moisture and the cracking of some small volatile molecules and functional groups. The main decomposition stage occurred between 350 and 520 °C. The maximum rate of weight loss (DTG_max_) appeared at approximately 420–485 °C, resulting from devolatilization reactions [27] and intensive pyrolysis processes. With increasing coal rank, the initial pyrolysis temperature shifted to higher values, and the total weight loss decreased noticeably. Furthermore, the DTG curves indicate that the peak temperature moved toward higher temperatures as coal rank increased.

#### 3.1.2. FTIR Analysis

**Figure 5:**
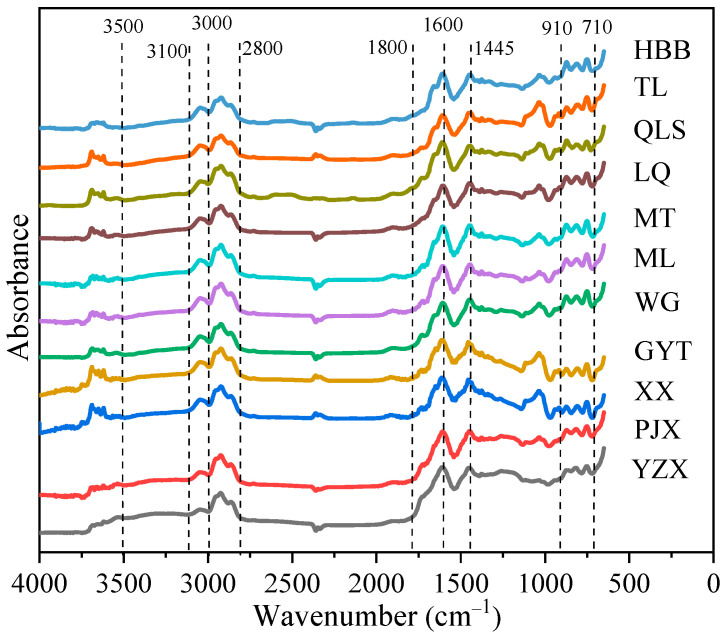
FTIR spectra of coals samples.

The FTIR spectra of the raw coal samples are shown in Figure 5. It can be observed that all coals exhibited similar spectral profiles. A notable feature common to each spectrum was the distinct variation in bands. The band in the range of 2800–3100 cm^−1^ was assigned to aliphatic C–H stretching vibrations, while the region between 3000 and 3100 cm^−1^ corresponds to aromatic C–H stretching vibrations [28]. Bands appearing between 1800 cm^−1^ and 1000 cm^−1^ are primarily attributed to oxygen-containing functional groups. Specifically, the band near 1600 cm^−1^ was ascribed to aromatic C=C stretching, and the band at 1445 cm^−1^ arises from the asymmetric deformation vibration of CH_3_. The spectral region between 910 cm^−1^ and 710 cm^−1^ was associated with out-of-plane deformation vibrations of aromatic C–H. Additionally, the absorption in the 3600–3700 cm^−1^ range was characteristic of clay minerals such as kaolinite [29]. Among the different coal samples, YZX coal showed greater intensity in the bands corresponding to functional groups, which was consistent with its higher content of oxygen-containing and aliphatic functional groups. The observed trend in oxygen content aligns with the results from ultimate analysis, and the distribution of aliphatic groups corresponds with coal rank.

### 3.2. XRD Analysis of Different Rank Coals During the Coking Process

#### 3.2.1. XRD Analysis

**Figure 6:**
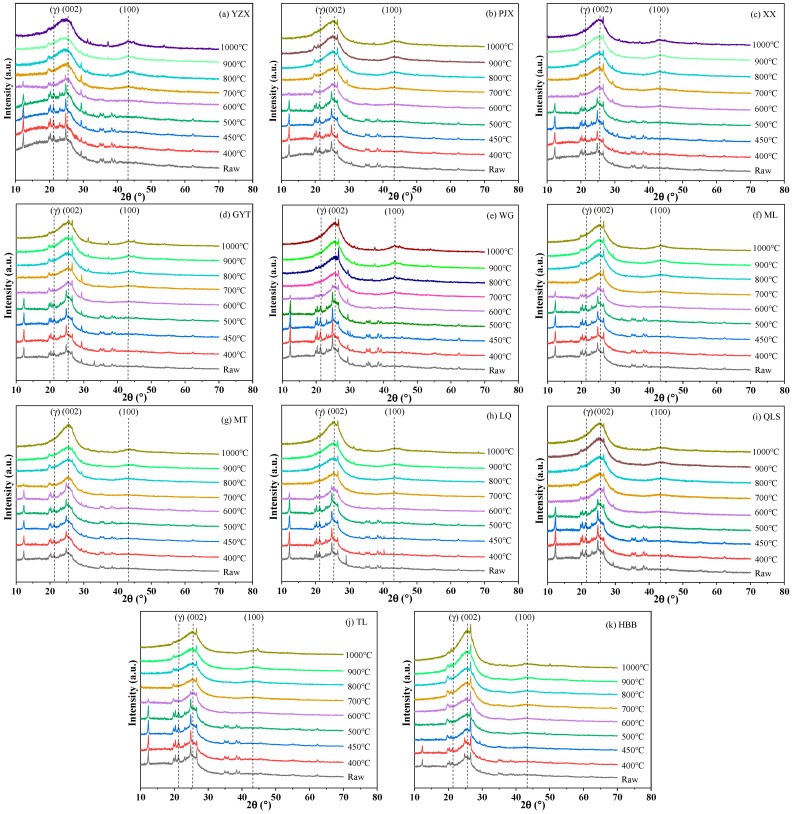
XRD analysis of different rank coals during the coking process.

The XRD analysis of different rank coals during the coking process is shown in Figure 6, which depicts eleven coal samples of different ranks tested at multiple temperatures. As shown in Figure 6, the γ peak (approximately at 2θ = 22°) corresponds to amorphous carbon in the carbon structure, indicating the stacking of alkyl side chains. The 002 peak (approximately at 2θ = 25°) represents the stacking structure of aromatic lamellae within the carbon microcrystalline framework, reflecting both the degree of directional alignment and the stacking height of aromatic layers. The 100 peak (approximately at 2θ = 43°) relates to the condensation degree of aromatic rings in the carbon structure and denotes the size of the aromatic lamellae [15,16]. It can be observed that the intensity of the 002 peak increases, while that of the γ peak decreases, with increasing coal rank and temperature.

#### 3.2.2. The Changes in the Carbon Structure of Different Rank Coals

As shown in Section 2.3.3, the γ, 002, and 100 bands of the different rank coals under different temperatures were obtained from the curve fitting, as shown in Figure 3, including the angle, FWHM, and area of the γ, 002, and 100 bands. The carbon structural parameters of L_c_, L_a_, d_002_, N, *n*, and f_a_ were calculated by Equations (1)–(6) from the results of curve fitting [22,23,24,30]. The XRD analysis results of eleven coals under different temperatures during the coking process were calculated. One sample of the structural parameters of peak fitting is shown in Table 3.

With the increase in temperature, the peak position of the fitted 002 peak gradually shifted to the right, the peak area increased, and the half-peak width decreased, so the 002 peak deformation became sharper, the peak intensity increased, and the carbon microcrystalline aromatic structure increased. The FWHM and area of the γ peak decreased with temperature, which meant the amorphous carbon in the carbon structure shifted into the regular carbon structure [16,31].

**Figure 7:**
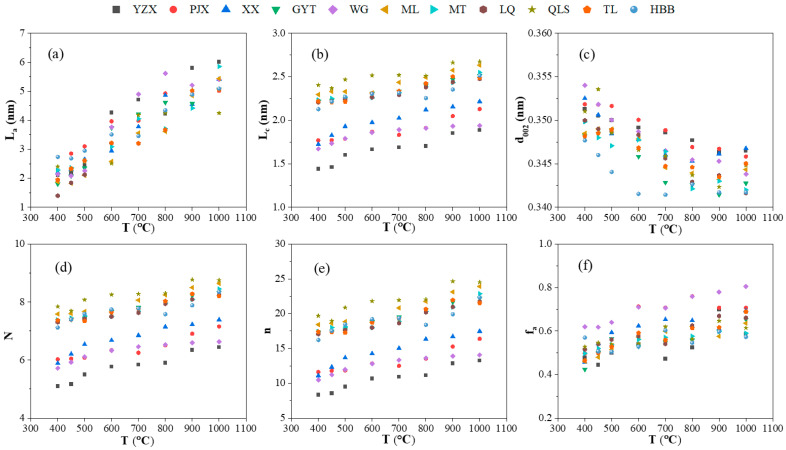
XRD parameters of samples under different temperatures with different ranks during the coking process: (**a**) L_a_; (**b**) L_c_; (**c**) d_002_; (**d**) N; (**e**) *n*; (**f**) f_a_.

The correlations between the carbon structural parameters of L_c_, L_a_, d_002_, N, *n*, f_a_, and temperature with different ranks during the coking process are shown in Figure 7. It was found that the structural parameters of the eleven coals changed consistently. With the increased temperature, the carbon structural parameters L_a_, L_c_, N, *n*, and f_a_ of coking coal were increased, and the opposite trend was observed for d_002_. This illustrated that the diameter of aromatic lamellae became larger, the spacing between aromatic layers decreased continuously, and the number of aromatic lamellae also increased, leading to an increase in the height of aromatic layers. It indicated that the coal molecules undergo condensation reactions to form larger aromatic structures during the pyrolysis process. The d_002_ decreased, which it meant that the coke gradually transformed into a graphitized structure. With the increase in temperature, the aromatic lamella height (L_c_) showed an upward trend during the coking process.

Figure 7a showed that the aromatic cluster size (L_a_) of the coal samples increased with rising temperature. Above 400 °C, coal decomposition commenced, leading to a noticeable rise in L_a_. Beyond 500 °C, the L_a_ values of various coals increased significantly. This result indicated that the molecular structure of coal achieved solidification after softening, melting, flowing, and swelling, forming a larger molecular planar structure during the semi-coke formation stage [5,8]. The aromatic lamella diameter L_a_ of the samples increased rapidly between 600 and 800 °C, indicating that condensation reactions following solidification promote the transformation of small molecular structures into larger aromatic systems [32]. This process expands the planar carbon aromatic framework and enlarges the aromatic nuclei, which is attributed primarily to polycondensation reactions accompanied by H_2_ release within this temperature range [10]. As a result, smaller aromatic rings condense to form larger ones, thereby increasing L_a_ [33]. Regarding coals of different ranks, the aromatic lamella diameter of high-rank coal coke was slightly smaller, whereas that of low-rank coal appeared marginally larger in the later coking stage. Furthermore, with increasing coal rank, the aromatization rate slowed.

Figure 7b shows the carbon structural parameter of aromatic lamella stacking height (L_c_) of raw coals and the heated samples during coking processing. For different raw coals, L_c_ was around 2 nm, as the literature has reported [34]. L_c_ was increased from 1.35 to 2.32 nm, with a rank that meant the condensation of aromatic carbon was compact [5]. Changes in the carbon structures of different rank coals were slight. It could be seen that the value of L_c_ increased with the increase in temperature, and its aromatic structure increased. Below 450 °C, the coals started to pyrolyze, and L_c_ hardly changed. When the molecular structure of coal reached the stage of solidified semi-coke formation after 500 °C, L_c_ increased slightly. The L_c_ of the eleven coal samples rose slowly at 600–800 °C. L_c_ rose rapidly after 800 °C, and the coal condensed into coke, with increased ordering of carbon structures at higher temperatures [35]. At this stage, the crosslinking reaction led to the increased height of the aromatic nuclei. For coals with low ranks, the layer of aromatization was less than for the high-rank coals. Samples YZX and XX, with low ranks, showed greater changes in L_c,_ though the coke properties of these samples were not very good. The L_c_ of coking coals expanded more slowly than for other coal samples. This meant that samples with good coal properties showed minor changes in L_c_. Changes in L_c_ were too great, leading to deterioration in coke properties. As coal rank increased, L_c_ value also increased, indicating greater structural ordering.

It can be seen in Figure 7c that the aromatic lamella spacing (d_002_) of coal samples showed a decreasing trend with an increase in temperature. This indicated that the aromatic structure became more compact and the connection between layers was closer [33,35]. The d_002_ of coals is increased slightly at 400 °C because of softening and melting. With the increase in temperature, the aromatic lamella spacing decreased. After 800 °C, the d_002_ of the samples changed slightly. The decrease in d_002_ reflected that the lamella spacing gradually reduced, and the orderliness of the structure was improved when the coke temperature reached above 900 °C. The lamella spacing of the coke of low-rank coal was larger than that of high-rank coal. This indicated that the graphitization degree of coal increased during the coking process, and the defective amorphous carbon structure transformed into a more ordered graphitized carbon structure.

As shown in Figure 7d, the number of aromatic lamellae (N) in carbon structures exhibited gradual increasing trends during the coking process, indicating a growth in aromatic structures and an enhancement in cross-linking within the carbon matrix. The aromatic lamellae count rose with increasing coal rank: low-rank raw coal contained approximately five lamellae, whereas high-rank coal averaged about seven. For coke, the average number of aromatic lamellae ranged from six to nine [31,33]. Specifically, coke derived from low-rank coal contained six–seven lamellae, while that from coking coals exceeded eight, reflecting the superior coking properties of the latter. During coking, the number of aromatic lamellae (N) increased, whereas the interlayer spacing (d_002_) showed no significant change or only a slight decrease. Consequently, the crystallite stack height (L_c_) demonstrated an overall upward trend, suggesting a positive correlation between Lc and N. This correlation implies that cross-linking reactions were promoted by the increase in stacked aromatic layers. From Figure 7e, it can be observed that the number of aromatic rings (*n*) within a single aromatic lamella also increased with temperature. As temperature rose, aromatic clusters in the coal samples expanded through continuous condensation reactions within the lamellae, leading to greater numbers of aromatic rings per lamella and improved structural ordering of the carbon material.

It can be seen from Figure 7f, with the increase of temperature, that the aromaticity (f_a_) of coal structure showed an upward trend during the pyrolysis and coking process. Between 400 and 500 °C was mainly the stage where coking coal underwent thermal decomposition due to the decomposition of aliphatic chains in coal. Many aliphatic side chain structures and oxygen-containing functional groups were broken and were released as small molecular gases and tar [15,36]. The reductions in aliphatic structures resulted in increases in aromaticity. Solid matter solidified into semi-coke after 500 °C, while after 600 °C, aromaticity increased significantly, which was because coal released small molecular aliphatic structures, which polymerized into larger aromatic structures. The number of aromatic structures increased, and f_a_ gradually increased during the coking process. When the pyrolysis temperature was above 800 °C, secondary polycondensation reactions occurred, which accelerated the process of ordering the carbon structure of semi-coke. The reason was that the carbon order was more regular at high temperatures, so increased aromatic carbon content gradually increased aromaticity (f_a_) [37]. The structure of coal transformed into coke and became more compact [38].

**Figure 8:**
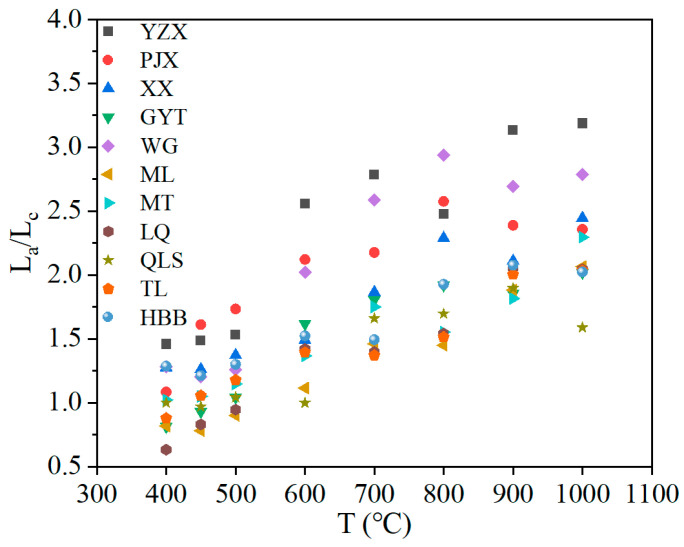
Correlation between L_a_/L_c_ and the temperature of samples during the coking process.

During the coking process, the crystal structure of coke showed a growth trend characterized by an increase in transverse size, number of aromatic lamellae, and longitudinal size, and a gradual decrease in the spacing between aromatic lamellae. The ratio of L_a_/L_c_ represents the change in the proportion of the lateral and longitudinal dimensions of the microcrystalline structure [14]. Figure 8 shows the ratio of L_a_/L_c_ of coke within this temperature range. According to the change in L_a_/L_c_, it could be found that the growth in the lateral dimension of the microcrystalline structure of the coke was larger than that of the longitudinal dimension. It was expanded approximately twice as much as raw coal. The main reason was that the condensation of the aromatic structure occurred during coke formation. The diameters of the aromatic rings of different rank coals were expanded by various degrees. The condensation reaction generated large amounts of H_2_, increasing the lateral dimensions of the aromatic layers [10]. The cross-linking reactions at high temperatures caused the condensation of aromatic nuclei to form more layers. Thus, the cross-linking density and the number and height of the aromatic layers increased.

### 3.3. Changes in Carbon Structure Parameters with Coal Rank During the Coking Process

#### 3.3.1. Relationship Between Coal Rank and L_c_ During Coke Formation

**Figure 9:**
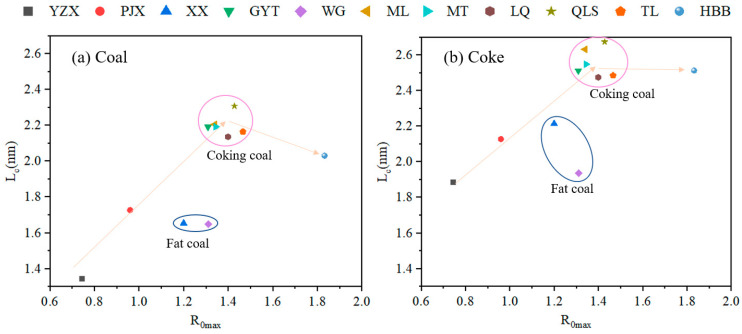
Correlation between coal rank and L_c_ of samples under different temperatures during the coking process: (**a**) all the raw coal samples; (**b**) all the cokes.

Changes in carbon structure parameters with coal rank during coking pyrolysis were studied. To understand the coke properties, the correlations between the carbon structure parameters of the coke properties and the coal rank were analyzed. XRD was used to analyze the structural parameters of L_a_, L_c_ from coal to coke during coke formation. The correlation between the coal rank and L_c_ of samples under different temperatures during the coking process showed a positive relationship, as shown in Figure 9.

From Figure 9a,b, it can be seen that the L_c_ and coal rank showed an upward trend, with an increase in coal rank for the different coals and cokes. For the raw coals, the L_c_ of height was between 1.344 nm (YZX) and 2.309 nm (QLS). The L_c_ values of the cokes were between 1.885 and 2.685 nm. The increase in L_c_ was 0.541 and 0.8 nm for YZX and QLS, respectively. The L_c_ values of coking coals (GYT, ML, MT, LQ, QLS, and TL) were the largest, followed by lean coal (HBB), 1/3 coking coal (PJX), fat coal (XX, WG), and gas coal (YZX). The L_c_ values of fat coals (XX and WG) were not too large, and less than 1/3 coking coals. The L_c_ of high-rank coal (HBB) was lower than that of coking coal. L_c_ always increased with the increasing temperature of the coking process, as shown in Figure S1. The height of the aromatic layer of coke increased slightly after reaching the resolidification temperature. L_c_ obviously increased when the temperature was above 900 °C. Changes in L_c_ were evident for all coals during the coking process.

#### 3.3.2. Relationship Between Coal Rank and L_a_ During Coke Formation

**Figure 10:**
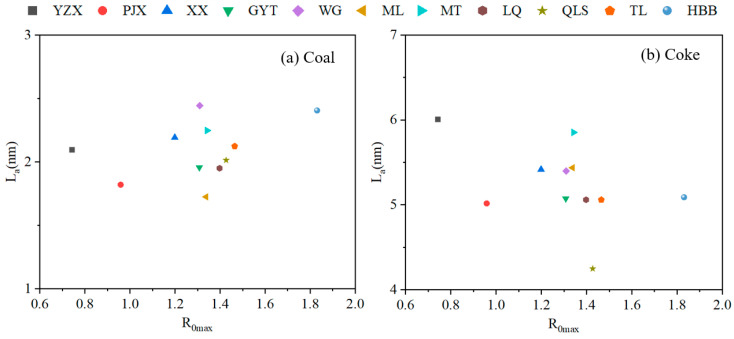
Correlation between coal rank and L_a_ of samples under different temperatures during the coking process.

Figure 10 shows the correlation between coal rank and L_a_ for samples under different temperatures during the coking process. The L_a_ values of coal samples were between 1.725 and 2.405 nm. The data are consistent with the literature data [14]. Whereas the L_a_ of coke increased greatly, the L_a_ of coal was about three times larger than that of coke. It can be seen that the transverse sizes significantly expanded during the evolution of coal to coke. This meant the transverse size of the aromatic layer changed greatly because of the condensation reaction during coke formation.

### 3.4. Changes in Carbon Structures with Coke Properties During the Coking Process

**Figure 11:**
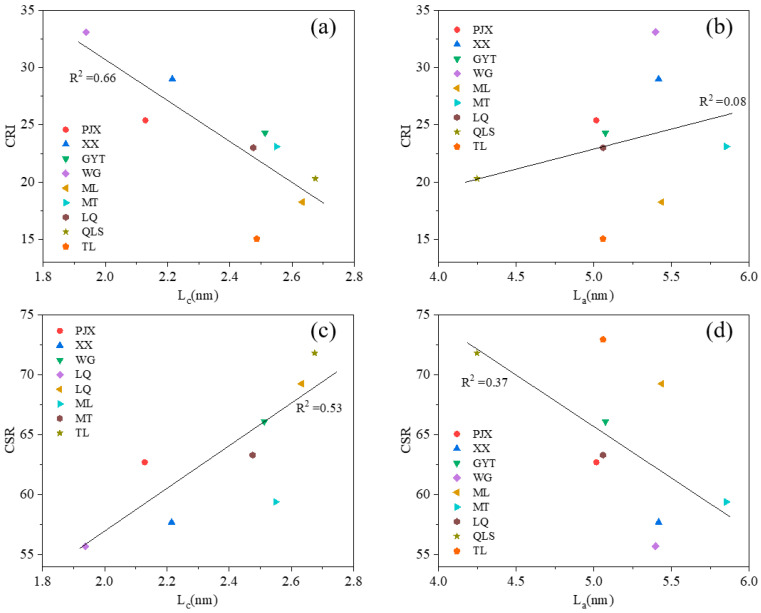
Correlation between CRI/CSR and L_c_/L_a_ of samples under different temperatures during the coking process: (**a**) L_c_ and CRI; (**b**) L_a_ and CRI; (**c**) L_c_ and CSR; (**d**) L_a_ and CSR.

The relationship between coke properties (CRI/CSR) and carbon microstructure (L_c_ or L_a_) was investigated using nine mid-rank coals, as shown in Figure 11. Two samples, namely the low-rank coal YXZ and the high-rank coal HBB, were excluded from the analysis due to their markedly distinct CRI/CSR characteristics. A negative correlation was observed between L_c_ and CRI, indicating that coke quality improved with increasing L_c_ values. Figure 11a showed that CRI was inversely proportional to L_c_, with a correlation coefficient (R^2^) of 0.66. Samples with L_c_ values below 2.4 nm exhibited CRI values exceeding 25%, suggesting that lower Lc is detrimental to coke properties. Conversely, when L_c_ exceeded 2.67 nm, coke properties were also adversely affected. As shown in Figure 11c, CSR exhibited a positive correlation with L_c_, yielding an R^2^ value of 0.53, which is consistent with findings reported in the literature [9,22]. Samples with L_c_ values below 2.4 nm displayed CSR values lower than 65%, whereas those with L_c_ values above 2.4 nm generally possessed higher coke strength.

The relationship between L_a_ and CRI was positive in Figure 11b, which meant the coke properties were bad, with the increase of L_a_ depending on the correlation coefficient being poor. As shown in Figure 11b, the L_a_ of samples showed high quality within the range of 5 to 5.5 nm, though they were all within this range. Figure 11d showed that the CSR was negative with L_a,_ and the R^2^ was 0.37. Samples WG and XX showed poor quality coke, since their CSR values were below 60, as shown in Figure 11d. On the other hand, some samples with L_a_ within the range of 5.0 to 5.5 nm had CSR values above 65. Such samples showed high-quality coke since they had high crystallite size and also high coke strength. The L_a_ showed poor quality if the L_a_ of coke was too small or too big. For bituminous coals, the changes in carbon structures were significant during the formation of coke for all coals.

The correlations between the coal ranks and L_a_ values of samples under different temperatures during the coking process is shown in Figure S2. After 900 °C, the L_a_ values of all the samples clearly increased, especially for low-rank coals.

Figure S3 illustrates the correlations between the CRI values and the aromatic layer diameters (L_a_) of samples at various temperatures during the coking process. As temperature increased, the aromatic layer diameters exhibited gradual expansion. Above 600 °C, the increases in L_a_ became less pronounced. Furthermore, L_a_ demonstrated a clear positive correlation with coal rank. Beyond 900 °C, all samples showed a marked rise in L_a_, particularly for coals with low CRI (indicative of higher-quality coke). This suggests that superior coke quality corresponds to a relatively modest L_a_ value. In general, a smaller L_a_ is associated with better coke quality. These observations imply that a slightly reduced aromatic layer diameter may correspond to a lower CRI, thereby reflecting improved coke quality.

Correlation between the CRI and L_c_ values of samples under different temperatures during the coking process is shown in Figure S4. With an increase in temperature, the height of the aromatic layer increased slightly. The aromatic layer height of the coke was relatively low. Therefore, the CRI of the coke was high and its coke properties were poor. The coke with a larger L_c_ had a condensed coke molecular structure and better coke properties. This indicated that the height of the aromatic layers had an impact on the coke properties. The higher the stacking height, the better the coke properties. This was consistent with lower CRI values and better coke properties.

The correlations between CSR and the diameters of the aromatic layers (L_a_) of the samples at different coking temperatures are presented in Figure S5. As temperature increased, L_a_ exhibited a gradual expansion. Below 500 °C, the aromatic layer diameter remained largely constant. Beyond 600 °C, a moderate increase in L_a_ was observed. Notably, in cokes with inferior properties, the rise in La occurred at an earlier stage. After 900 °C, L_a_ values for all samples increased significantly, indicating considerable growth in the aromatic layer diameters. Conversely, samples with relatively small L_a_ diameters tended to demonstrate better coke properties. These results suggest that a moderately small L_a_ may contribute to higher CSR values and improved coke quality.

Figure S6 illustrates the correlation between the Coke Strength after Reaction (CSR) and the stacking heights (L_c_) of aromatic layers in samples subjected to different temperatures during the coking process. With increasing temperature, the diameter of the aromatic layers expanded gradually. In contrast, the variation in L_c_ with temperature was not pronounced, although a slight increase in layer height was observed. L_c_ demonstrated a positive correlation with CSR, indicating that greater stacking heights correspond to higher coke strength and improved coke properties. These results suggest that enhanced stacking height of aromatic layers is associated with elevated CSR values, which in turn reflects superior coke quality.

### 3.5. Raman Analysis of Different Rank Coals During the Coking Process

#### 3.5.1. Raman Analysis

**Figure 12:**
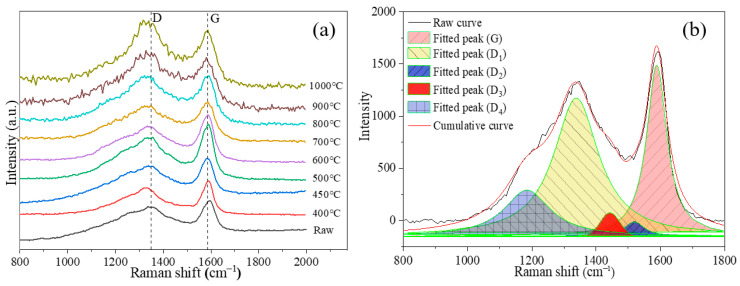
Raman spectra of QLS coal at different temperatures: (**a**) Raman spectra of QLS coals; (**b**) peak fitting of QLS coal at 700 °C.

Figure 12a shows the Raman spectra of the QLS sample at different temperatures. Two distinct vibration bands were observed near 1350 cm^−1^ and 1590 cm^−1^, corresponding to the D band and G band, respectively [4]. The G band was attributed to the E^2^_2g_ vibration mode of the ideal graphite lattice, which was caused by the breathing mode of aromatic rings in amorphous carbon materials. The D band is associated with the A_1g_ vibration mode and originates mainly from structural defects, edge disorder and low-symmetry carbon arrangements within the graphite lattice [4,25]. In coal molecular structures, the D band reflects planar defects and disordered domains. In the raw Raman spectra, the D and G bands consistently exhibit overlapping features. To gain further insight into the carbon skeleton structure and to elucidate microstructural evolution during the coking process, peak fitting of the Raman spectra was conducted to extract latent structural details.

Following the band assignments for coal, Raman spectra are summarized in Table 2. The spectra were deconvoluted using Origin 2021 into four Lorentzian peaks (G, D_1_, D_2_, and D_4_) and one Gaussian peak (D_3_), in accordance with methods reported in the literature [4,25]. The R^2^ values were above 0.95. A representative peak-fitting result is displayed in Figure 12b. It can be seen the additional bands induced by defects in the microcrystalline lattice appear in the 800–2000 cm^−1^ region, including peaks at approximately 1185 cm^−1^ (D_4_), 1380 cm^−1^ (D_1_), 1465 cm^−1^ (D_3_), 1540 cm^−1^ (D_2_), and 1590 cm^−1^ (G). The band position and assignment of these bands are summarized in Table 2.

#### 3.5.2. Changes in the Structure Parameters of Different Rank Coals

**Figure 13:**
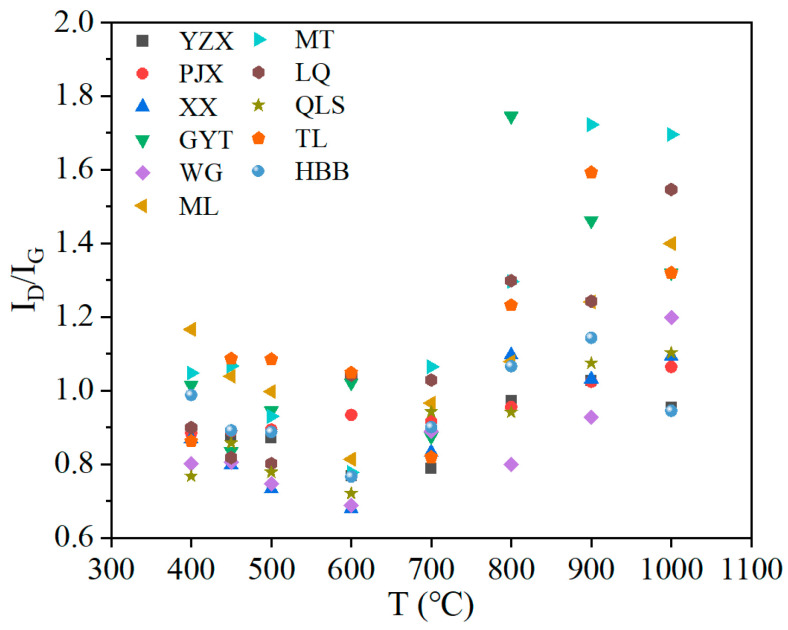
The changes in I_D_/I_G_ of different samples with pyrolysis temperature.

The changes in I_D_/I_G_ with the pyrolysis temperature of different samples are shown in Figure 13. Figure 13 shows the variation trends in I_D_/I_G_ of different coals with pyrolysis temperature. The D bands in the Raman spectra correspond to the existence of structural defects or disorders in the graphite layers of coal. The G bands correspond to the stretching of sp^2^-bonded carbon atoms in the graphite layers. I_D_/I_G_ represented the relative amount of large disordered aromatic rings (*n* ≥ 6), which is an important parameter for characterizing the degree of order in carbon materials. As shown in Figure 13, coal was decomposed gradually after 400 °C and I_D_/I_G_ decreased at below 600 °C. The breaking of weak bonds during pyrolysis was the main cause of the breakage of sp^2^-bonded carbon atoms in coal. As the temperature increased, the carbon structural defects increased and the broken side chains were deposited on the surface of coal char, increasing its amorphousness. When the temperature was above 600 °C, I_D_/I_G_ increased, which meant the formation of defects and amorphous structures, leading to a decrease in the order of coal char [39].

## 4. Discussion

**Figure 14:**
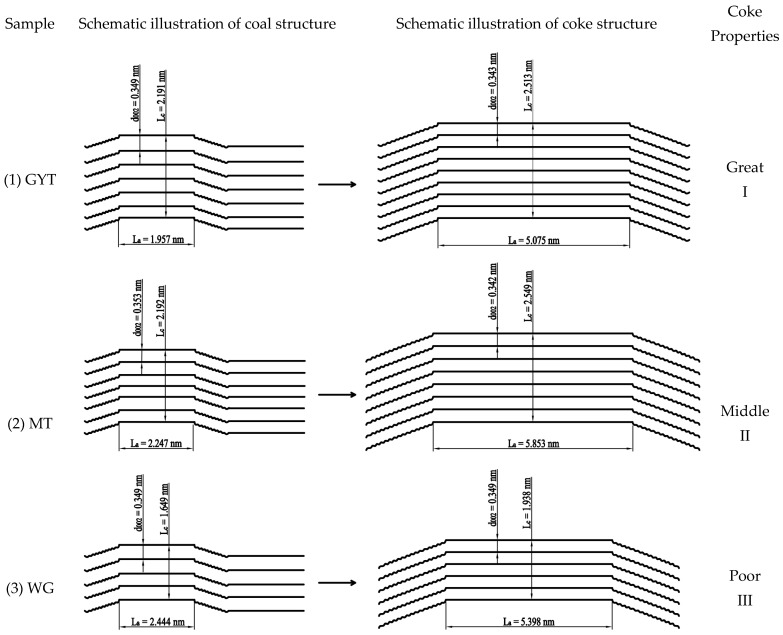
Schematic illustration of the changes in the structural parameters of coking coals during coal-to-coke transformation.

Figure 14 shows a schematic illustration of the changes in the structural parameters of coking coal during coal-to-coke transformation; the correlation between the structural properties of coke and the L_c_ and L_a_ structural mechanisms of formation from coal to coke. The strength of the coke was mainly determined by the microstructure. During the coking process of coal, a series of complex physical and chemical changes, such as softening, melting, polycondensation, and solidification, form the basic structural units of graphite-like microcrystals [2]. These units were connected by strong covalent bonds into a continuous and anisotropic three-dimensional network structure. During the coking process, the carbon structure of each layer was stacked through π-π bonds to form larger and roughly parallel aromatic ring layers [31,40]. When multiple layers were stacked in parallel, the structure of graphite-like microcrystals was formed [40,41], as illustrated in the structural diagram of coke in Figure 14. The carbon microcrystals of coke were different from perfect graphite crystals with a completely parallel structure. Although the layers were parallel, the relative positions between layers were randomly offset and lacked the long-range ordered stacking of graphite. Despite this “disordered layer structure”, the carbon atoms within each layer were connected by extremely strong covalent bonds (such as C–C sp^2^ bonds), which provided coke with extremely high strength [17,33]. The strength of individual microcrystals was high. However, if the connected bonds between the carbon structures were weak, they would break easily. The covalent bond connection between microcrystals was the fundamental reason for the mechanical strength of the coke.

The main purpose of the technologies used to improve the strength of coke (such as coal blending, coking, coal preheating, etc.) was to optimize the coking process to promote the formation of larger and better-oriented graphite-like microcrystals. It was important to improve the covalent bond connections between microcrystals, and the size and quantity of defects such as pores and cracks decreased [17]. The number and strength of chemical covalent bond connections were the key to distinguishing high-strength coke and low-strength coke. Ultimately, coke showed high mechanical strength in terms of anti-fracture and anti-wear on a macroscopic scale. Therefore, the coke was able to withstand the huge pressure of the burden column and intense friction in the blast furnace without pulverizing [1].

As shown in Figure 14, the L_c_ values of GYT and MT coke showed larger microcrystal structures. The proportion of inert basal planes increased, while the growth of edge lengths was relatively slow. This leads to a decrease in the proportion of active sites, resulting in a low index of CO_2_ reactivity (CRI) of the coke and strong resistance to reactions with CO_2_. Although the L_c_ values of GYT and MT coke were similar, the L_a_ of MT coke was wider, so its coke properties were worse than those of GYT coke. It can be seen that the L_c_ size of the WG coke was the smallest. This illustrated that the coke with a large unit mass had a larger specific surface area and longer total edge length. Therefore, the coke directly exposed more active carbon atoms, providing a large number of attack points for CO_2_. Thus, the WG coke showed high CRI and poor coke properties. Therefore, the L_c_ of the coke showed a good correlation with the CRI. The coke with a smaller L_c_ showed a high CRI value and poor reactivity. The coke with a larger L_c_ had a low CRI value, which meant the coke had good reactivity.

The value of the Coke Strength after Reaction (CSR) depended on the structure of the remaining carbon framework after the reaction. It was mainly closely related to the number of aromatic layers (N). The more aromatic layers, the higher the CSR. The fewer aromatic layers, the worse the CSR.

For polymer materials, the larger the molecular weight, the higher the strength [42,43]. The lower the molecular weight, the poorer the strength. For coal or coke, they were a kind of polymer material. It was still suitable for the theory that high molecular weight or large carbon structure showed heavy strength.

## 5. Conclusions

The coke samples were prepared at varying coking temperatures using a one-sided heating furnace. The coking behavior and carbon structure parameters of the coals were investigated. The relationships between carbon structure parameters and the properties of both the raw coal and coke were analyzed during the coke formation process. The main conclusions are as follows.

As temperature increased, L_a_, L_c_, N, *n*, and f_a_ were increased, while d_002_ decreased, indicating compaction and a trend toward graphitization of the carbon structure. Changes in the carbon structure of different rank coals correlated positively with increasing rank, L_c_ showed an upward trend with an increase in coal rank.

It was found that the ratio of L_a_/L_c_ was increased, with the lateral dimension of the coke microcrystalline structure expanding more significantly than the longitudinal dimension. The increase in L_a_/L_c_ was approximately twice that of raw coal. The cross-linking reactions occurring at elevated temperatures, promoting condensation of aromatic nuclei and leading to a greater number of aromatic layers. Both the quantity and height of these aromatic layers were enhanced as a result of the cross-linking process.

A negative correlation was observed between L_c_ and CRI, indicating that higher L_c_ values correspond to improve coke quality. The correlation coefficient (R^2^) was 0.66. In contrast, CSR showed a positive correlation with L_c_, with a correlation coefficient of 0.53. When the L_c_ value exceeded 2.4 nm, coke quality was considered favorable, characterized by a CRI below 25% and a CSR above 65%. However, a slight degradation in coke quality was noted when L_c_ increased beyond 2.67 nm.

The L_a_ values of the samples varied within the range of 5 to 5.5 nm and the coke process showed higher coke strength due to the larger grain sizes of the samples. Conversely, when the L_a_ value of the coke was beyond the range of 5 to 5.5 nm, the coke strength was considerably reduced. Compared with other coal samples, the carbon microcrystal structure of bituminous coals exhibited more pronounced changes during the formation of coke. Furthermore, coke derived from higher molecular mass precursors demonstrated superior strength.

## Figures and Tables

**Table 1:** Proximate analysis, ultimate analysis, and properties of coal samples.

		YZX	PJX	XX	GYT	WG	ML	MT	LQ	QLS	TL	HHB
Categories		GC	1/3CC	FC	CC	FC	CC	CC	CC	CC	CC	LC
Proximate analysis (wt%, ad)	M	2.23	1.30	0.97	0.61	0.4	0.89	0.72	0.9	0.94	0.51	0.93
	A	7.89	8.87	9.28	10.23	9.91	9.19	9.33	8.94	9.96	10.23	11.51
	V	32.49	31.30	29.09	20.44	22.94	22.67	20.93	18.86	19.74	19.8	13.82
	FC	57.39	58.53	60.66	68.72	66.75	67.25	69.02	71.3	69.36	69.46	73.74
Ultimate analysis (wt%, daf)	C	88.52	85.78	87.29	84.28	90.91	86.79	90.5	88.75	86.63	88.4	93.02
	H	4.71	4.68	4.5	3.06	4.27	5.61	3.94	3.78	3.82	5.25	4.07
	N	1.92	1.39	1.79	2.03	1.69	2.13	1.76	1.15	1.89	1.75	1.58
	S	0.47	0.36	0.64	1.34	0.8	0.91	0.5	1.25	0.39	1.29	0.37
	O *	4.38	7.78	5.79	9.29	2.33	4.56	3.3	5.06	7.27	3.31	0.96
R_o max_		0.744	0.959	1.199	1.308	1.31	1.338	1.343	1.398	1.427	1.465	1.831
Coke properties	M_40_	65.2	84.0	82.4	82.4	85.2	90.9	84.8	84.4	87.6	89.3	/
	M_10_	27.2	8.0	8.8	6.8	9.2	6.3	8.4	8.8	7.2	6.7	/
	CRI	59.4	25.4	29	24.3	33.1	18.25	23.1	23	20.3	15.1	/
	CSR	23.6	62.7	57.7	66.1	55.7	69.25	59.4	63.3	71.8	73.0	/
Coke grade		-	II	III	I	III	I	II	III	II	I	-

ad—air dried basis; daf—dry ash free. * Differential calculation.

**Table 2:** Assignment of Raman spectrum bands in coals [4,24,25,26].

Center/cm^−1^	Bands	Assignment	Band Type
1590	G	Aromatic ring quadrant breathing; alkene C=C	sp^2^
1540	D_2_	Amorphous carbon structures; aromatics with 3–5 rings	sp^2^
1465	D_3_	Aryl–alkyl ether; para-aromatics	sp^2^, sp^3^
1380	D_1_	C=C between aromatic rings and aromatics with not less than 6 rings	sp^2^
1185	D_4_	Caromatic=Calkyl; aromatic (aliphatic) ethers; C=C on hydroaromatic rings; hexagonal diamond carbon sp^3^; C-H on aromatic rings	sp^2^, sp^3^

**Table 3:** XRD analysis of the characteristic parameters of WG coals during coking.

	γ			002			100			L_c_/nm	L_a_/nm	d_002_/nm	N	*n*	f_a_
	2θ/°	FWHM	Area	2θ/°	FWHM	Area	2θ/°	FWHM	Area						
Raw	20.44	7.40	7250	25.22	5.16	9879	45.28	7.21	841	1.649	2.444	0.3532	5.7	10.3	0.5767
400 °C	20.41	6.35	5931	25.15	5.09	9687	44.81	8.20	951	1.671	2.144	0.3540	5.7	10.5	0.6203
450 °C	20.53	5.89	6107	25.31	4.91	9858	44.24	8.43	1104	1.732	2.083	0.3518	5.9	11.2	0.6175
500 °C	21.37	7.43	6056	25.45	4.75	10,758	44.71	7.79	969	1.791	2.255	0.3500	6.1	12.0	0.6398
600 °C	21.42	6.22	4387	25.55	4.58	10,724	44.07	4.67	696	1.859	3.758	0.3487	6.3	12.8	0.7097
700 °C	21.43	5.29	4725	25.71	4.50	11,335	43.44	3.58	1018	1.892	4.893	0.3465	6.5	13.4	0.7058
800 °C	21.43	5.02	4376	25.79	4.46	13,792	43.51	3.12	1132	1.910	5.615	0.3455	6.5	13.6	0.7591
900 °C	21.61	4.13	3858	25.80	4.41	13,637	43.53	3.36	1456	1.931	5.204	0.3453	6.6	13.9	0.7795
1000 °C	22.13	3.64	3738	25.91	4.40	15,405	43.39	3.24	1489	1.938	5.398	0.3438	6.6	14.1	0.8047

## Data Availability

The original contributions presented in this study are included in the article. Further inquiries can be directed to the corresponding authors.

## Supplementary Materials

The following supporting information can be downloaded at https://www.mdpi.com/article/10.3390/ma19010168/s1, Figure S1: Correlation between coal rank and L_c_ of samples under different temperatures during the coking process; Figure S2: Correlation between coal rank and L_a_ of samples under different temperatures during the coking process; Figure S3: Correlation between CRI and L_a_ of samples under different temperatures during the coking process; Figure S4: Correlation between CRI and L_c_ of samples under different temperatures during the coking process; Figure S5: Correlation between CSR and L_a_ of samples under different temperatures during the coking process; Figure S6: Correlation between CSR and L_c_ of samples under different temperatures during the coking process.
