# Supplementary material for: Correlations Between Carbon Structure and Properties by XRD and Raman Structural Studies During Coke Formation in Various Rank Coals
Source: Materials (Basel). 2026 Jan 2;19(1):168. doi: 10.3390/ma19010168 (PMC12787029; doi:10.3390/ma19010168)
Supplement: Supplementary file 1 [file materials-19-00168-s001.zip › materials-4013731-SA.pdf]

materials

MDPI

Article

Correlations between Carbon Structure and Properties by XRD and Raman Structural Studies During Coke Formation in Various Rank Coals

Lu Tian [1], Jinxiao Dou [1,*], Xingxing Chen [1], and Jianglong Yu [1,2,*]

[1] Key Laboratory of Advanced Coal and Coking Technology of Liaoning Province, School of Chemical Engineering, University of Science and Technology Liaoning, Anshan 114051, China; 320163300148@ustl.edu.cn (L.T.); xingchenstar79@163.com (X.C.)

[2] Suzhou Industrial Park Monash Research Institute of Science and Technology, and Southeast University-Monash University Joint Graduate School, Suzhou 215123, China

* Correspondence: doujx123@163.com (J.D.); jianglongyu@163.com; Tel.: +86-(0)412-5929105 (J.Y.)

Supplementary Materials: The following supporting information can be downloaded at: https://www.mdpi.com/article/10.3390/ma19010168/s1, Figure S1: Correlation between coal rank and L_c of samples under different temperatures during the coking process; Figure S2: Correlation between coal rank and L_a of samples under different temperatures during the coking process; Figure S3: Correlation between CRI and L_a of samples under different temperatures during the coking process; Figure S4: Correlation between CRI and L_c of samples under different temperatures during the coking process; Figure S5: Correlation between CSR and L_a of samples under different temperatures during the coking process; Figure S6: Correlation between CSR and L_c of samples under different temperatures during the coking process.

Received: 13 November 2025
Revised: 12 December 2025
Accepted: 19 December 2025
Published: 6 January 2026

Copyright: © 2026 by the authors. Licensee MDPI, Basel, Switzerland. This article is an open access article distributed under the terms and conditions of the Creative Commons Attribution (CC BY) license.

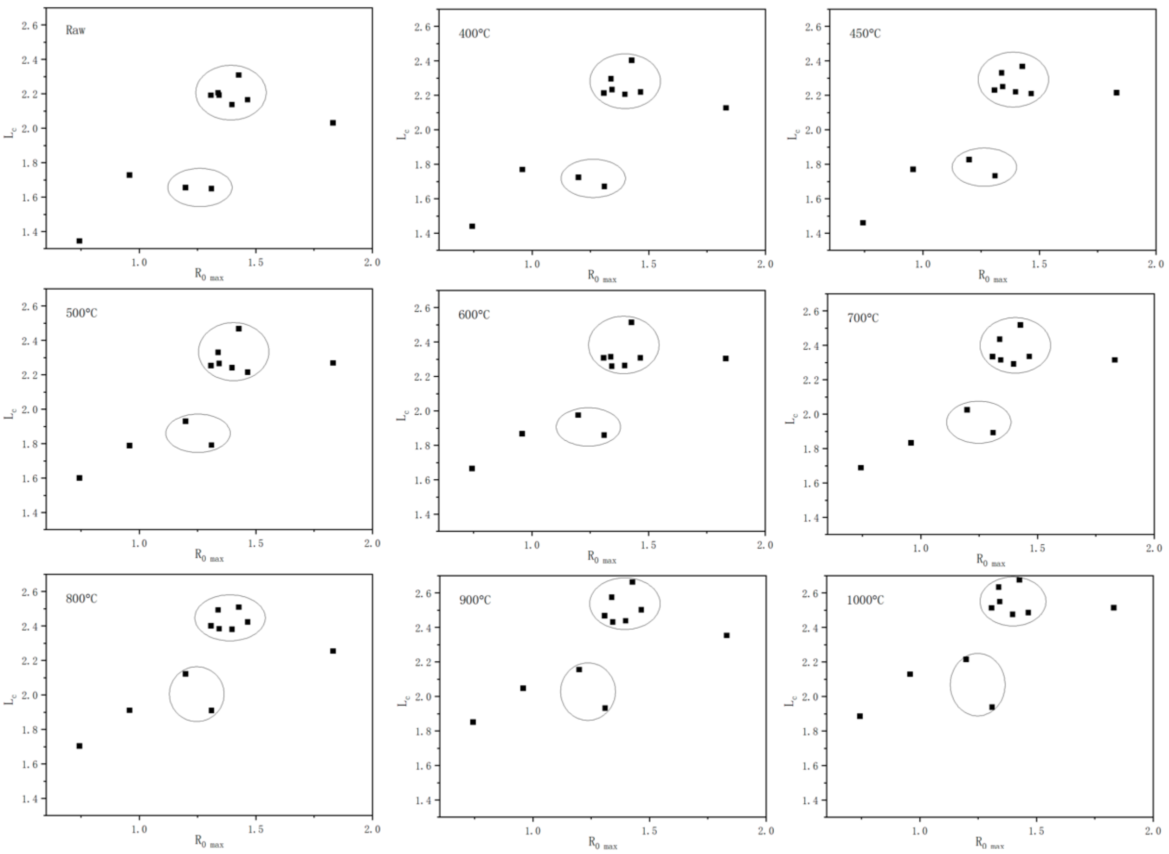

Figure S1. Correlation between coal rank and L_c of samples under different temperatures during the coking process

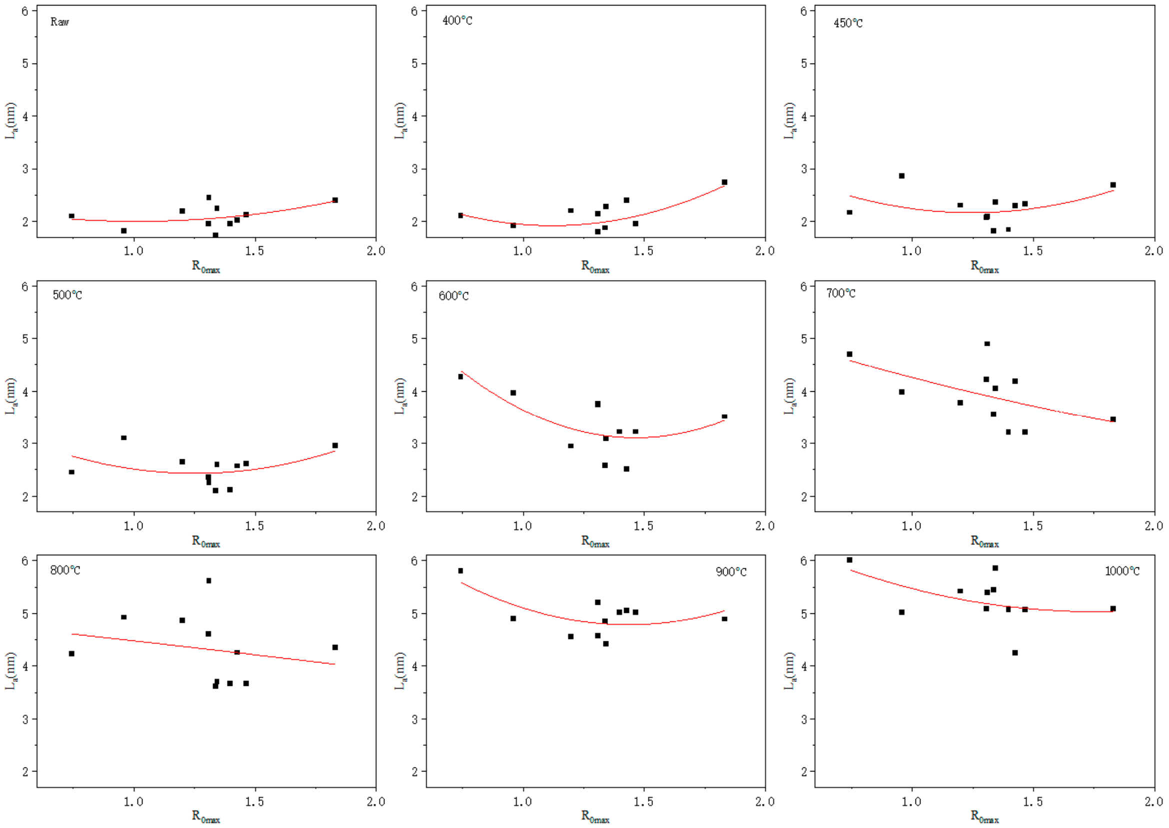

Figure S2. Correlation between coal rank and La of samples under different temperatures during the coking process

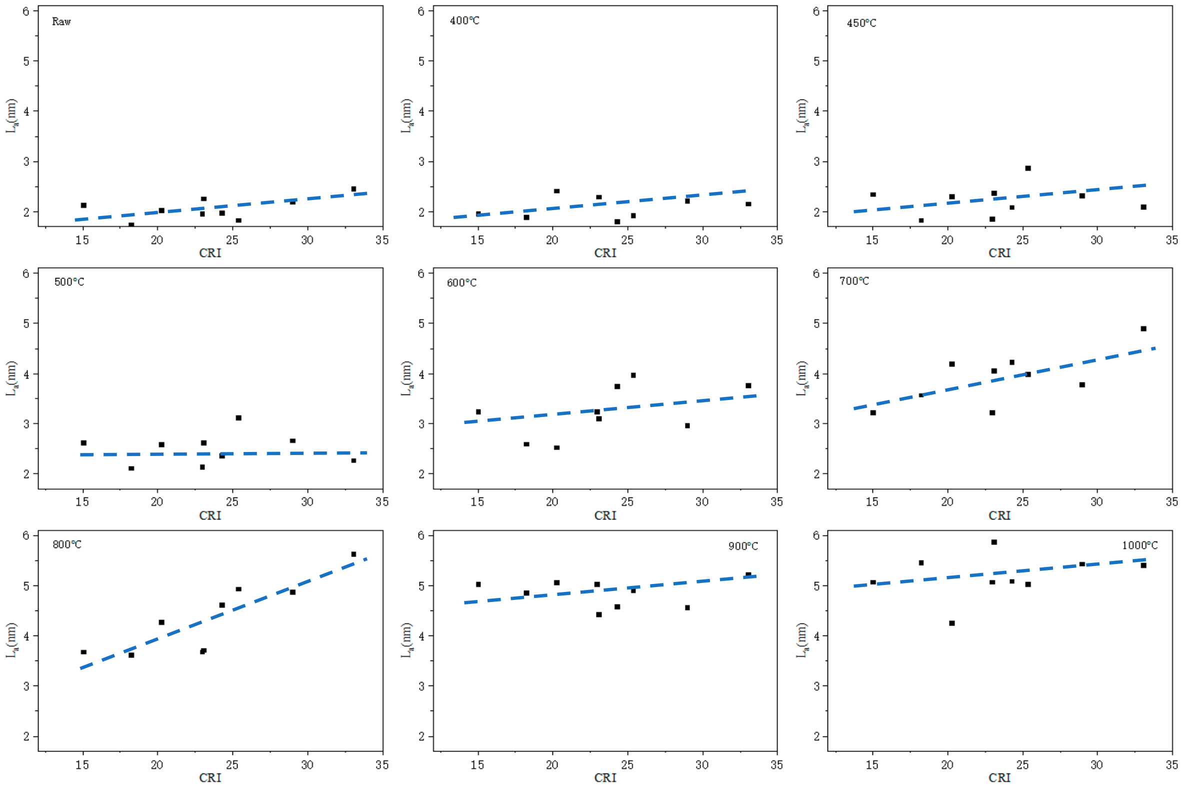

Figure S3. Correlation between CRI and L_a of samples under different temperatures during the coking process

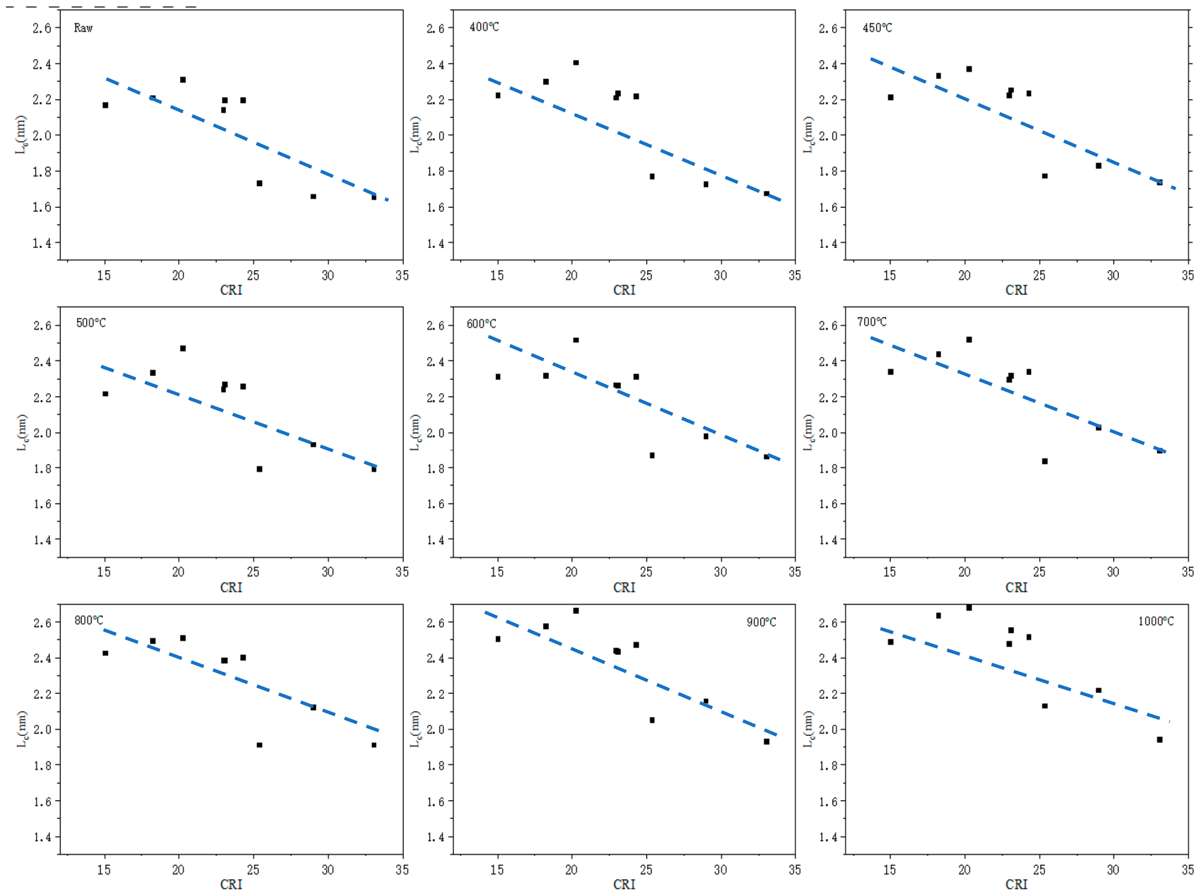

Figure S4. Correlation between CRI and L_c of samples under different temperatures during the coking process

https://doi.org/10.3390/ma19010168

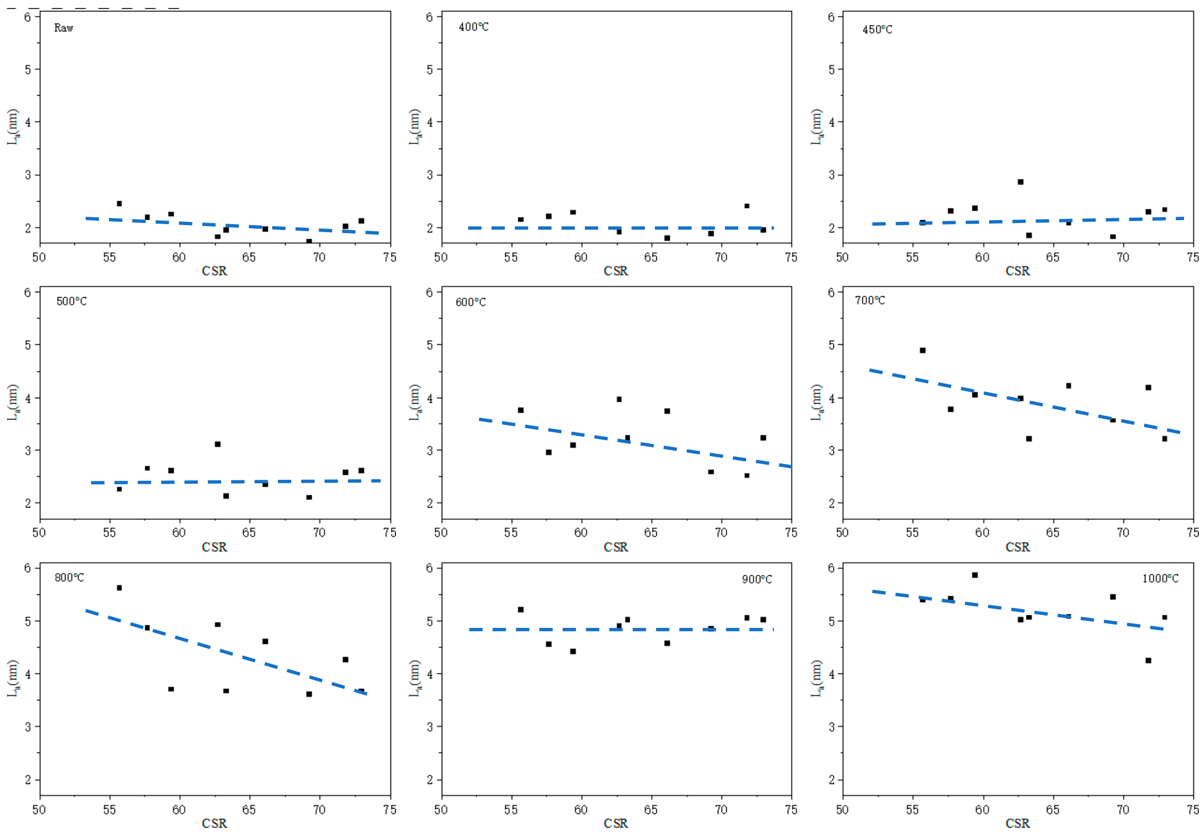

Figure S5. Correlation between CSR and L_a of samples under different temperatures during the coking process

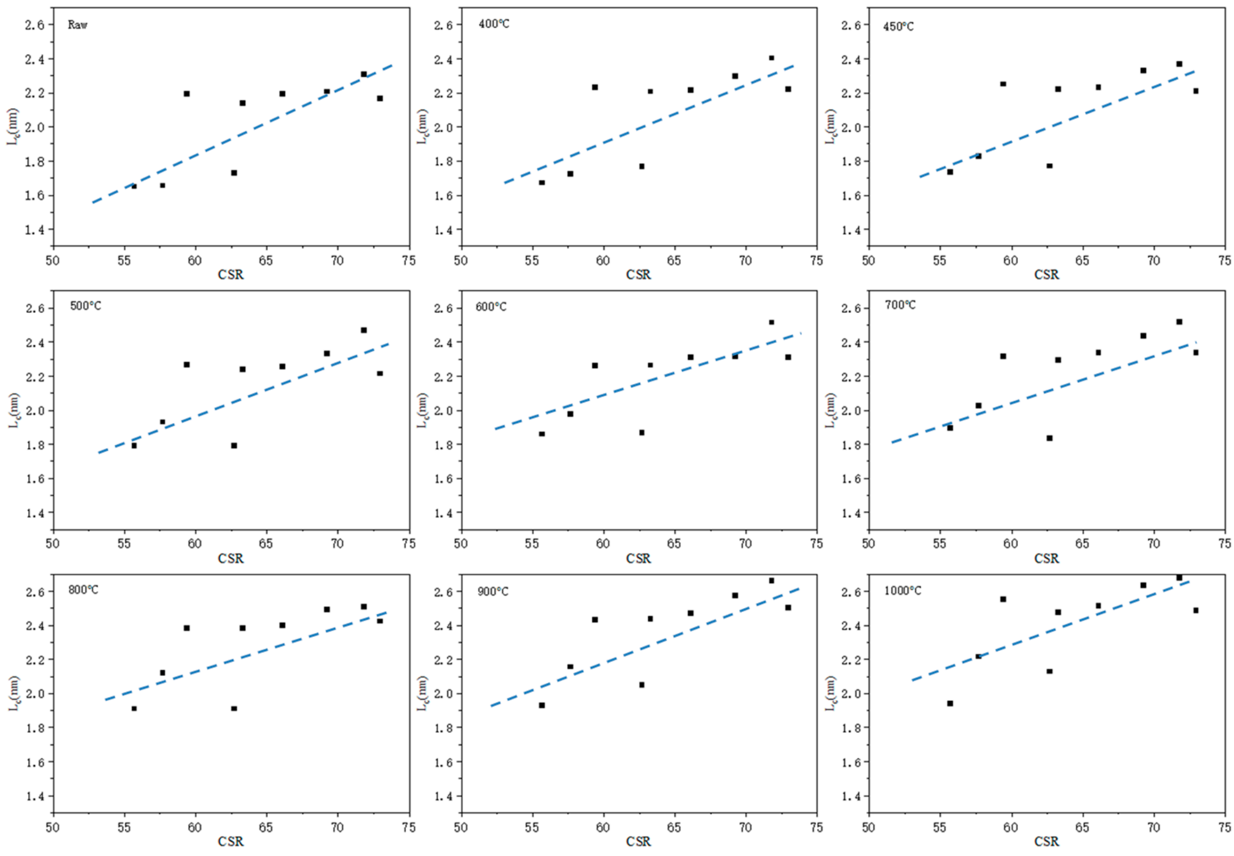

Figure S6. Correlation between CSR and L_c of samples under different temperatures during the coking process

https://doi.org/10.3390/ma19010168
